# Analytical validation of a prognostic prostate cancer gene expression assay using formalin fixed paraffin embedded tissue

**DOI:** 10.1186/s12920-018-0442-y

**Published:** 2018-12-27

**Authors:** Paul Wallace Medlow, Christopher James Steele, Andrena Marie McCavigan, Wesley Reardon, Christopher Michael Brown, Shauna May Lambe, Felipe Augusto Andre Ishiy, Steven Michael Walker, Gemma Elizabeth Logan, Olaide Yaqeen Raji, Viktor Berge, Betina Katz, Elaine Williamson Kay, Katherine Sheehan, Ronald William Watson, Denis Paul Harkin, Richard Darragh Kennedy, Laura Anne Knight

**Affiliations:** 1 Almac Diagnostics Ltd., Craigavon, Northern Ireland UK; 2 Centre for Cancer Research and Cell Biology, Queens University Belfast, Belfast, UK; 3 Department of Urology, Oslo University Hospital, Oslo, Norway; 4 Department of Pathology, Oslo University Hospital, Oslo, Norway; 5 Department of Pathology, RCSI, Beaumont Hospital, Dublin, Ireland; 6 UCD School of Medicine, Conway Institute, University College Dublin, Belfield, Dublin, Ireland

**Keywords:** Prostate cancer, Prognostic, Recurrence, Analytical validation, Metastatic

## Abstract

**Background:**

There is a clear need for assays that can predict the risk of metastatic prostate cancer following curative procedures. Importantly these assays must be analytically robust in order to provide quality data for important clinical decisions. DNA microarray based gene expression assays measure several analytes simultaneously and can present specific challenges to analytical validation. This study describes the analytical validation of one such assay designed to predict metastatic recurrence in prostate cancer using primary formalin fixed paraffin embedded tumour material.

**Methods:**

Accuracy was evaluated with a method comparison study between the assay development platform (Prostate Disease Specific Array) and an alternative platform (Xcel™ microarray) using 50 formalin-fixed, paraffin-embedded prostate cancer patient samples. An additional 70 samples were used to establish the assay reportable range. Determination of assay precision and sensitivity was performed on multiple technical replicates of three prostate cancer samples across multiple variables (operators, days, runs, reagent lots, and equipment) and RNA/cDNA inputs respectively using the appropriate linear mixed model.

**Results:**

The overall agreement between the development and alternative platform was 94.7% (95% confidence interval, 86.9–98.5%). The reportable range was determined to be 0.150 to 1.107 for core needle biopsy samples and − 0.214 to 0.844 for radical prostatectomy samples. From the precision study, the standard deviations for assay repeatability and reproducibility were 0.032 and 0.040 respectively. The sensitivity study demonstrated that a total RNA input and cDNA input of 50 ng and 3.5 μg respectively was conservative.

**Conclusion:**

The Metastatic Assay was found to be highly reproducible and precise. In conclusion the studies demonstrated an acceptable analytical performance for the assay and support its potential use in the clinic.

**Electronic supplementary material:**

The online version of this article (10.1186/s12920-018-0442-y) contains supplementary material, which is available to authorized users.

## Background

Prostate cancer is one of the most common cancers in men in the United States with an estimated 161,000 new cases of prostate cancer in 2017, accounting for 21% of all new cancers diagnosed [1]. Furthermore, prostate cancer represents 4% of all cancer deaths, with approximately 26,700 deaths due to the disease [1]. Despite the high cure rate that accompanies the use of active surveillance, radical prostatectomy or radiotherapy as initial therapies for localised prostate cancer, approximately 35% of patients experience biochemical recurrence, as defined by an increase in Prostate Specific Antigen (PSA) levels within 10 years of treatment. One third of these patients present with metastatic disease within 8 years of PSA elevation [2, 3]. Current assessment of metastatic risk includes tumour histopathological assessment (Gleason grading) and tumour staging following PSA elevation. However, these are often limited due to their prognostic variability [4] meaning that there is a clear requirement for additional prognostic strategies that estimate the likelihood of metastasis. The role of both single gene and molecular signature biomarkers in identifying cancer and predicting treatment response is increasing. The MammaPrint® Breast Cancer Recurrence Assay represents one of a number of commercially available tests with prognostic and/or predictive utility in breast cancer. These have paved the way for the emergence of additional tests focused in other disease indications including prostate cancer (Oncotype DX, Decipher®).

Almac Diagnostics Ltd. have developed a microarray-based assay that measures the expression of 70 genes (See Additional file 1: Table S1) to prospectively identify a molecular subgroup of prostate cancer patients at increased risk of metastatic disease recurrence following radical surgery or radiotherapy with curative intent. The 70-gene assay (known as the Metastatic Assay) has been defined as having an underlying biology driven by processes that promote prostate cancer progression and metastasis e.g. WNT signaling and FOXM1 regulation [5, 6].

Diagnostic core needle biopsies (CNB) or primary tumour resections followed by formalin fixation represent the primary source of tumour tissue in the prostate cancer setting and represent a significant challenge to molecular analysis due to limited tissue quantity, nucleic acid/protein cross-linking and nucleic acid degradation [7]. The Metastatic Assay has been developed on Almac’s formalin fixed paraffin embedded (FFPE) optimized Prostate Cancer Disease Specific Array (DSA) and classifies a patient sample as either having metastatic-like biology (assay positive) or non-metastatic (assay negative) based on a predefined threshold. DNA microarrays are a collection of oligonucleotide probes complimentary to target sequences and are used to quantify gene expression from a nucleic acid solution. In application, RNA is extracted from tissue, converted to labelled cDNA before being applied to the microarray where the target sequences bind to their complimentary probes in a process known as hybridization. The microarray is then washed and stained with fluorescently labelled streptavidin before being scanned and the strength of the fluorescent signal measured. The strength of the signal is dependent on the amount of target sequences bound to their complimentary probe(s). The presence of a large number of probes, representing thousands of genes on a single microarray is why DNA microarrays have long been used as a discovery tool to identify differences in gene expression between diseased and normal tissue, particularly in prostate cancer [8].

Using this platform, the Metastatic Assay has been clinically validated through two large independent retrospective clinical cohorts and *in-silico* analysis has been performed in three independent public microarray datasets [5, 6]. The assay demonstrates strong prognostic utility that could improve patient risk stratification and identify those patients that may benefit from treatment intensification (assay positive) from those patients that should be considered for active surveillance (assay negative).

For an assay to be considered suitable for use within clinical practice it must be analytically robust. Analytical validation of a DNA microarray assay can be challenging as several analytes are measured simultaneously, each with a different weighted effect on the assay algorithm and ultimately the final assay score. This study aimed to evaluate the analytical performance of the assay following the established guidelines published by the Clinical and Laboratory Standards Institute (CLSI). Although the analytical validation of other prostate cancer assays have been published previously, to the best of our knowledge this is the first evidence based analytical validation of such an assay on a cDNA microarray platform [9, 10]. The assay’s analytical accuracy, reportable range, precision and analytical sensitivity are reported.

## Methods

### Sample selection

A total of 60 prostate tumour FFPE CNB samples and 60 FFPE radical prostatectomy (RP) samples were used in the analytical validation studies. Specimens were sourced from multiple European sites including Oslo University Hospital, Cardiff University, University College Dublin and Northern Ireland Biobank (Queen’s University Belfast/Belfast Health and Social Care Trust). Overarching ethical approval for this study was obtained from the Health Research Authority (HRA) NRes Committee East of England (Norfolk) Research Ethics Committee (Ref: 15/EE/1066) with waived need for consent due to the retrospective nature of this study.

### Gene expression microarray profiling

All FFPE samples were sectioned into 5 × 5 μm sections and mounted on slides. The first slide was haematoxylin and eosin (H&E) stained and the region(s) of viable prostate carcinoma were marked by a board certified pathologist. Markings were transferred to the corresponding unstained slides and the tumour area was macrodissected into a microcentrifuge tube using a scalpel, in preparation for ribonucleic acid (RNA) extraction.

Total RNA was isolated using the Roche High Pure RNA Paraffin kit (Roche, Basel, Switzerland). Samples with a minimum concentration of 12.5 ng/μl, quantified using a Nanodrop Spectrophotometer (ThermoFisher, Santa Clara, CA), proceeded to cDNA generation using the Ovation FFPE WTA System kit (NuGEN Technologies Inc., San Carlos, CA). 3.5 μg of cDNA product was fragmented and labelled with the Encore Biotin Module (NuGEN Technologies Inc., San Carlos, CA) and hybridized overnight to the Almac Diagnostics proprietary Xcel™ microarray (Affymetrix, Santa Clara, CA). Each microarray underwent a series of washing and staining steps prior to being scanned on the Affymetrix 7G Scanner (Affymetrix, Santa Clara, CA). The resulting microarray data was pre-processed and a number of quality control (QC) steps were applied.

**Fig. 1:**
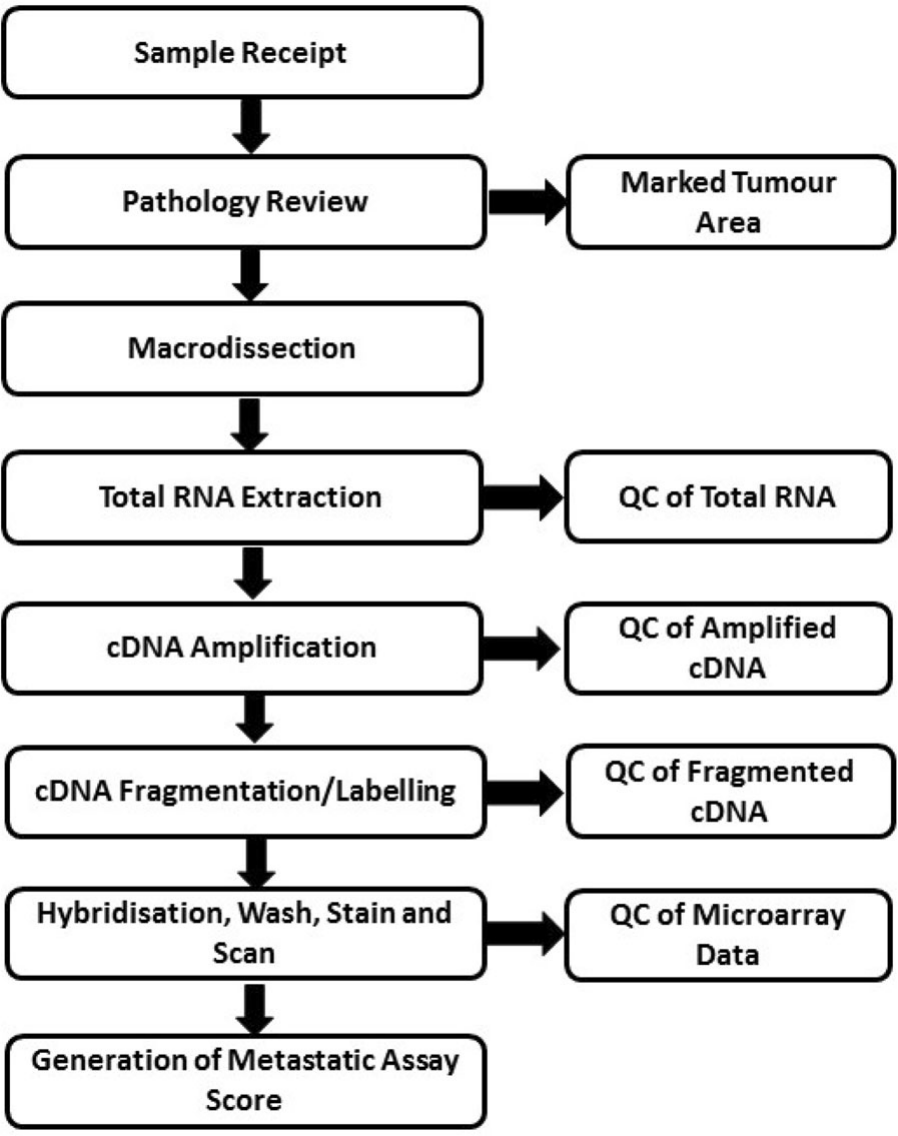
Overview of the Metastatic Assay workflow and quality control (QC)

Figure 1 outlines the sample workflow for the Metastatic Assay.

### Assay quality control

A number of QC steps were implemented during the protocol to ensure reliability of the assay performance (Fig. 1). The initial QC begins at pathological review followed by pathologist guided macrodissection. Samples were excluded if there were no viable tumour cells present. Secondly, total RNA concentration was assessed following extraction and a minimum concentration of 12.5 ng/μl of total RNA was required. Following amplification, cDNA yield was measured and a minimum yield of 3.5 μg was required. cDNA fragment length was assessed using capillary gel electrophoresis to confirm the presence of a fragment length profile typical of an FFPE sample. These QC limits were determined based on the manufacturer’s input requirements for the NuGEN Ovation FFPE WTA amplification kit and Encore Biotin Module respectively. The performance of the cDNA fragmentation reaction was assessed using the Agilent 2100 Bioanalyzer system to ensure at least 80% of cDNA transcripts are ≤200 nt in length prior to microarray hybridization. The hybridization controls *BioB, BioC, BioD* and *Cre* were applied to the cDNA prior to hybridization and assessed for their presence and expressing at increasing intensity. Finally, a range of QC metrics were applied to microarray data including array image analysis (AIA) for detection of surface and background artefacts, assessment of percent present, array intensity distribution analysis and principal component analysis. A percent present value below the QC limit of 15% disqualified a specimen from further analysis. Further detail is provided in the Additional file 2: Supplementary Methods. Assay scores were calculated as previously described [6].

An FFPE ES-2 cell line (ATCC, England, UK), serving as a gene expression control, was included in each processing batch from extraction through to microarray profiling. Almac Diagnostics have developed a gene expression signature with pre-defined limits of acceptance for this gene expression control sample ((mean target signature score 0.3542 +/− 3 standard deviations (SD)). These limits have been established over multiple analytical runs within Almac Diagnostics. This signature score was then used as control limits for the FFPE ES-2 cell line. In addition, a Universal Human Reference (UHR) RNA (Agilent Genomics, Santa Clara, CA) sample was included in each amplification batch. Each UHR was assessed using surveillance charts established through > 500 analytical runs. These charts monitor the Affymetrix parameters percent present and average signal absent. Westgard rules were applied to determine any violations outside of the mean +/− 3*SD [11]. An assay positive or UHR control that breached the pre-defined limits would result in repeat testing of all controls and batch of specimen samples.

### Analytical accuracy

The assessment of analytical accuracy for a given test is often determined through the quantification of systemic error between the candidate method and a reference method [12]. Due to the novelty of the assay, no reference method was available. In line with the CLSI EP09-A3 guideline, a method comparison study was performed as a surrogate for analytical accuracy to calculate the agreement in assay outcome between the development platform (Prostate DSA™, Affymetrix, Santa Clara, CA) and an alternative platform (Almac Xcel™ microarray, Affymetrix, Santa Clara, CA) [12]. The Prostate DSA and the Almac Xcel microarray contain 137,066 and 126,787 probe sets respectively. These probe sets target 19,944 unique gene transcripts in the Prostate DSA and 20,406 in the Xcel array. 19,465 of these genes overlap between the two array types of which the 70 genes targeted by the Metastatic Assay are included. RNA was isolated from 55 prostate tumour FFPE CNB (*n* = 15) and RP (*n* = 40) samples followed by microarray genomic profiling on both platforms. A selection of samples (10 CNB, 10 RP) were processed in duplicate giving a total sample number of 75 samples profiled on each platform. The assay was applied to each sample and the strength of agreement was estimated by the overall percent agreement in assay outcome between the development and Xcel™ microarray platforms. The positive percent agreement and negative percent agreement, both with respect to the development platform, were also calculated. Furthermore, exact 95% binomial confidence intervals were calculated for each measure of agreement. The flexibility of the Metastatic Assay to migrate to alternative technology platforms was further assessed on the Nanostring nCounter® platform and an RNA sequencing platform (Roche NimbleGen SeqCap RNA Targeted kit and Illumina MiSeq® Sequencer). Both platforms have the ability to quantify gene expression albeit through different chemistries. RNA sequencing uses next generation sequencing technology to sequence short strands of cDNA, aligning them back to a reference genome and counting the number of aligned reads. Alternatively Nanostrings’ nCounter® technology uses microscopic imaging to detect and count transcripts that have been hybridized to complimentary probes containing a unique barcoded identifier. These technologies overcome some of the disadvantages of the DNA microarray by being able to quantify both low and high expressed transcripts that may not be detected by DNA microarray technology due to background noise and hybridization saturation respectively. The correlation of assay scores and agreement in assay call between the development platform and both Nanostring and RNA sequencing platforms were calculated (See Additional file 2: Supplementary Methods).

### Reportable range

The reportable range of an assay is described as “the range of test result values over which the laboratory can establish or verify the accuracy of the instrument, kit, or test system measurement response” and encompasses the full range of reportable values for a given assay [13]. Our sample set represented only a subpopulation of prostate cancer samples. As such the lower and upper limits of assay range could not be determined. Therefore the reference sample group was used to establish the reference interval for each sample type (CNB and RP) as a surrogate for the assay reportable range.

A total of 120 prostate tumour FFPE samples were analysed using the assay. Sample selection incorporated a cohort of prostate cancer specimens from RP (*n* = 60) and a separate cohort of prostate cancer specimens from radical radiotherapy CNB (*n* = 60) which were identified by ensuring a balanced range of clinical characteristics (See Additional file 1: Table S2) and a representative distribution of Metastatic Assay scores from previous gene expression profiling. To minimise the influence of technical variability, samples were processed by a single operator using single reagent lots. Following the recommendations as outlined by the CLSI EP28-A3C guideline for sample numbers < 120 the robust method of estimation was used to determine a 95% reference interval for each sample type [14]. These are defined as the intervals that contain the central 95% of the assay scores for CNB samples and RP samples. In addition, 90% bootstrap confidence intervals were also calculated for the estimated lower and upper limits of the 95% reference intervals. A t-test was used to calculate any difference in mean Metastatic Assay score between CNB and RP samples.

### Analytical precision

A single site experimental design based on the CLSI guideline EP05-A3 was implemented to analytically validate assay precision [15]. This study was designed to measure intra-assay and inter-assay precision by introducing a number of variables including operator (*n* = 2), days (*n* = 20), runs (2 per day), critical reagent lots (*n* = 3), thermal cyclers (*n* = 2) and scanners (*n* = 2). This study used three pooled RNA samples, generated from either FFPE CNB (*n* = 1) or RP (*n* = 2) prostate cancer tissue, to comprise a sample set that represented each assay output (assay positive (CNB), assay negative (RP)) and a sample with an assay score close to the sample type specific medical decision point (RP). An assay positive FFPE ES-2 cell line sample was included within each run to identify assay score limits for use as a Metastatic Assay positive control in future clinical sample testing. Each of the three RNA pooled samples and the ES-2 FFPE cell line sample were tested in duplicate during each run at the nominal assay input of 50 ng. Assay variability due to reagent lots and equipment was assessed by randomly assigning one of two lots of reagents (Ovation FFPE WTA System kit, Encore Biotin Module and Xcel™ microarrays) and one of two pieces of equipment (thermal cyclers and Affymetrix 7G Genechip scanners) to each operator for a given run. A total of 40 valid runs were completed, 20 by each operator.

Based on the 20 × 2 × 2 study design (days x runs per day x replicates per run) where replicates are nested within runs, runs within days and equipment, reagent lot and operator nested within a run, a nested linear mixed model was used to estimate the sources of variability [16]. All components (day, run, equipment, reagent lot and operator) were treated as random effects. The formula for the linear mixed effects model implemented was:

$$ {\mathrm{Y}}_{\mathrm{i}\mathrm{j}\mathrm{klmn}}=\upmu +{\mathrm{D}}_{\mathrm{i}}+{\mathrm{R}}_{\mathrm{j}\left(\mathrm{i}\right)}+{\mathrm{E}}_{\mathrm{k}\left(\mathrm{i}\mathrm{j}\right)}+{\mathrm{L}}_{\mathrm{l}\left(\mathrm{i}\mathrm{j}\right)}+{\mathrm{O}}_{\mathrm{m}\left(\mathrm{i}\mathrm{j}\right)}+{\upvarepsilon}_{\mathrm{n}\left(\mathrm{i}\mathrm{j}\right)} $$

, *where:*

*Y*_*ijklmn*_: observed Metastatic Assay score for the *n*th replicate, by operator *m,* reagent lot *l*, equipment combination *k*, in the *j*th run, on the *i*th day.

*μ*: mean Metastatic Assay score.

*D*_*i*_*:* between-day variability.

*R*_*j (i)*_: between-run variability nested in day.

*E*_*k (i j)*_: between-equipment variability nested in run, which is nested in day.

*L*_*l (i,j)*_: between-lot variability nested in run, which is nested in day.

*O*_*m (i,j)*_: between-operator variability nested in run, which is nested in day.

ε_*n (i,j)*_: within-run (between-replicate) variability.

Bootstrap confidence intervals were derived for the parameters of the linear mixed effects model while confidence intervals for the total SD estimates were estimated using the method of Satterthwaite [17]. Repeatability was quantified by the within-run SD and reproducibility was quantified by the total SD, calculated by taking the square root of the sum of all variance component point estimates. Analytical imprecision is most prominent when assay scores are close to the medical decision point (threshold). A misclassification assessment, based on the analytical precision study and reportable range study data, was performed to estimate the effect of assay imprecision on clinical sample call. To perform this misclassification assessment, the estimated reproducibility of the threshold scoring sample was used to represent the imprecision of the Metastatic Assay. In particular, a normally distributed error using the total SD of the threshold scoring sample was added to the assay scores of the samples processed as part of the determination of the reference intervals and an assay call was made based upon the updated assay score. By comparing the assay call before and after the introduction of the assumed imprecision error, the proportion of samples that were misclassified due to imprecision was determined.

### Analytical sensitivity

The lower limit of quantification (LLOQ), described as “the lowest actual amount at which the analyte is reliably detected”, is used to define the analytical sensitivity of an assay [18]. However, evaluation of microarray sensitivity is complicated by the large number of separate analytes evaluated simultaneously and limited by cross hybridization and sources of biochemical and instrumentation noise [19, 20]. As such, treating the analyte of the assay as the total RNA, sensitivity was determined by assessing the effect of different total RNA inputs on assay score. Using three pooled RNA samples, representing each assay output (assay positive (CNB), assay negative (RP)) and a sample close to the sample specific medical decision point (RP), total RNA inputs were assessed both at the specification input (50 ng) and outside (12.5 ng, 25 ng and 100 ng) the specifications of the Ovation FFPE WTA System amplification kit. As an additional measure, the assay score was also assessed following manipulation of cDNA input. Using the same three pooled RNA samples, cDNA was generated and input into the fragmentation process at both the nominal assay input (3.5 μg) and outside (0.88 μg, 1.75 μg and 7 μg) the specifications of the Encore Biotin Module. Each sample was processed in triplicate using a single lot of assay reagents. A linear mixed effects model was used to investigate the effect of varying the level of RNA and cDNA input on assay score. In particular, estimates were calculated comparing the assay score at non-specification total RNA input amounts (12.5 ng, 25 ng and 100 ng) and cDNA input amounts (0.88 μg, 1.75 μg and 7 μg) to the assay score at the nominal total RNA input amount (50 ng) and cDNA input amount (3.5 μg) respectively.

## Results

### Analytical accuracy

**Fig. 2:**
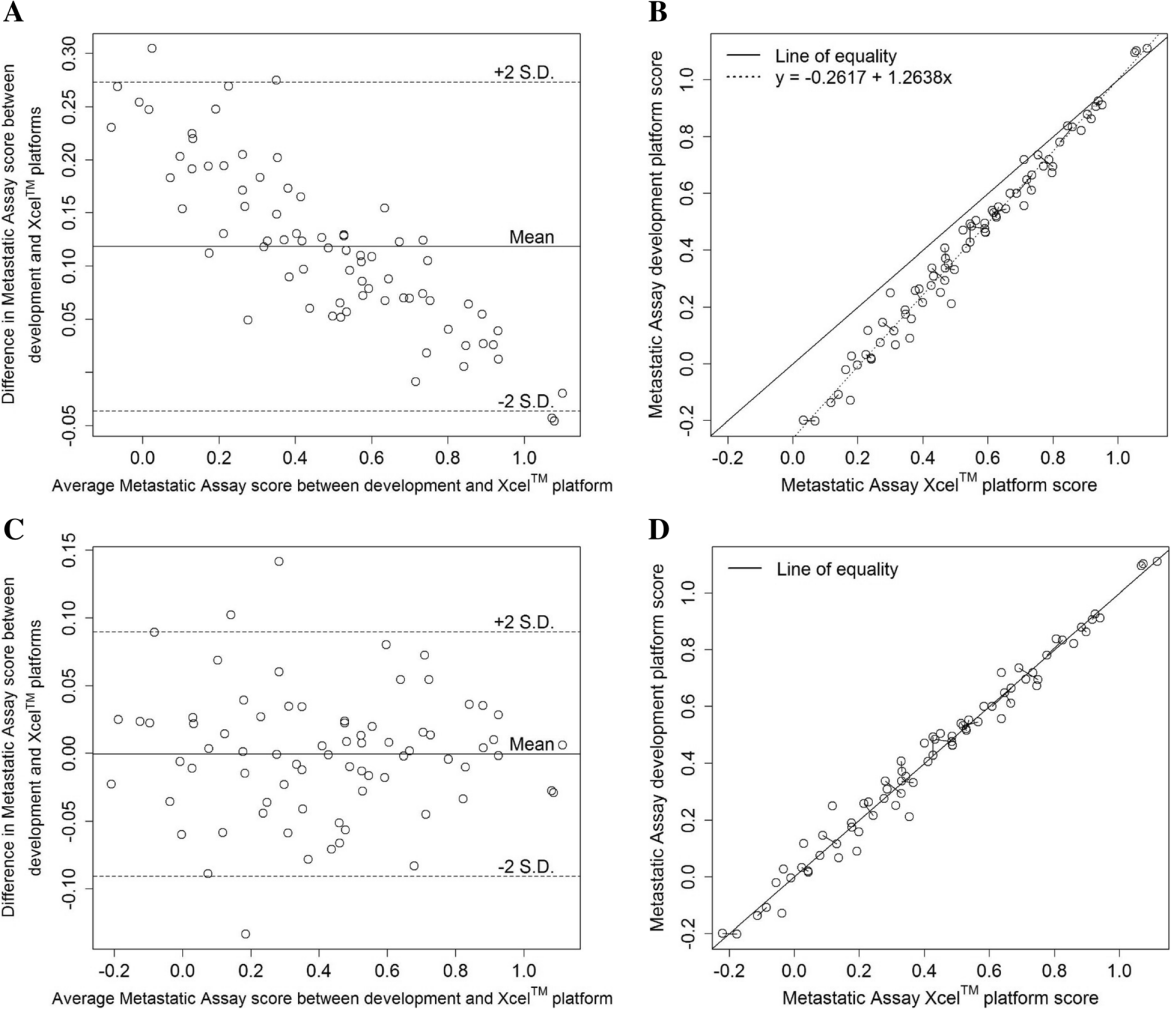
Bland-Altman plot of Metastatic Assay scores on the development and Xcel™ microarray platforms prior to (A) and post (C) linear bias correction. Scatter plot of Metastatic Assay scores from prostate cancer patients (*n* = 55) processed on the development and Xcel™ microarray platforms prior to (B) and post (D) linear bias correction. The dotted line is the estimated regression line. The solid line is the line of equality. Each open circle represents the assay score of the same sample profiled on the development and Xcel™ microarray platforms. Open circles connected by a solid line represent replicate samples

75 samples, incorporating 50 unique patient samples, were profiled on the development platform (Prostate DSA™) and on the alternative platform (Xcel™ array) for the analytical accuracy study. All samples and process controls profiled satisfied the QC criteria. A Bland-Altman plot was used to visualise the bias between the assay scores on both platforms (Fig. 2a) where the assumption of a constant bias between the platforms was found to be violated following a test of constant difference (*p* < 0.05) [21]. To account for the non-constant bias, a linear bias correction, given by the line of regression through the paired assay score observations, was applied to successfully migrate the assay between the platforms (Fig. 2b) (See Additional file 2: Supplementary Methods). After applying the linear bias correction, agreement between the platforms was estimated using the coefficient of individual agreement (CIA) [22] and satisfied the pre-defined criteria (*δ* = 0.78 < 1.24). The removal of the non-constant bias can also be observed from the post linear bias correction Bland-Altman plot (Fig. 2c) and scatter plot of paired assay scores (Fig. 2d). The overall percent agreement in assay call between the Prostate DSA™ and the Xcel™ microarray platforms was 94.7% (calculated using results displayed in Table 1) (95% CI, 86.9–98.5%). Furthermore, the positive and negative percent agreements were calculated as 91.7% (95% CI, 77.5–98.2%) and 97.4% (95% CI, 86.6–99.9%) respectively. The results of the migration and analytical accuracy analyses indicated the suitability of performing the analytical validation studies on the Xcel™ microarray platform, after applying the linear bias correction to the Metastatic Assay scores. A high degree of correlation in assay scores and overall percent agreement in assay calls was also demonstrated with both the Nanostring nCounter® platform (Pearson correlation *r* = 0.94 (See Additional file 1: Figure S1) and overall percent agreement = 94.2% (see Additional file 1: Table S3A and S3B)) and the RNA sequencing platform (Pearson correlation *r* = 0.94 (See Additional file 1: Fig. S2) and overall percent agreement = 87.9% (See Additional file 1: Table S4A and S4B)).

**Table 1:** Agreement in Metastatic Assay outcome between the development and alternative platforms

		Alternative Platform (Xcel™ Array)		
		Assay Positive	Assay Negative	Total
Development Platform (Prostate DSA™)	Assay Positive	33	3	36
	Assay Negative	1	38	39
	Total	34	41	75

*Abbreviations: DSA* = disease specific array

### Reportable range

**Fig. 3:**
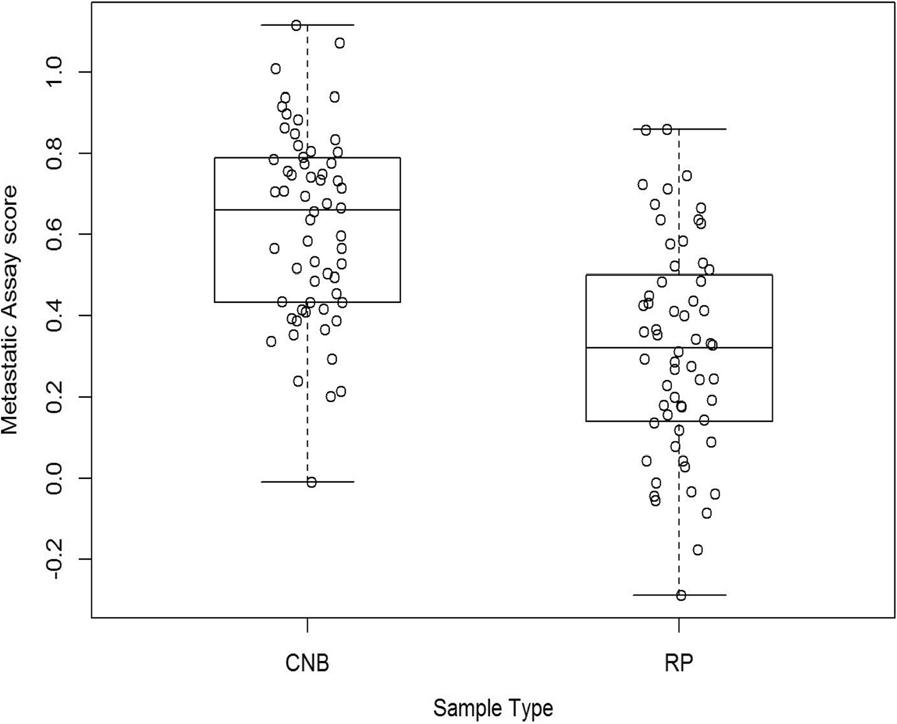
Box plots showing the distribution of the Metastatic Assay scores for core needle biopsy (CNB) and radical prostatectomy (RP) samples. Each open circle represents the assay score of each sample profiled on the Xcel™ microarray platform

Sixty prostate cancer CNB and 60 RP samples were used to establish sample type specific reference intervals (Fig. 3). Table 2 summarises the 95% reference intervals for CNB and RP samples. Also displayed are the 90% confidence intervals for the estimated lower and upper limits of the 95% reference intervals. The 95% reference interval limits for CNB and RP samples were calculated as (0.150, 1.107) and (− 0.214, 0.844) respectively. A significant difference (ANOVA *p* < 0.001) in mean Metastatic Assay scores between CNB (mean = 0.6216) and RP (mean = 0.3159) samples was noted. Differences in the distribution of DDRD scores was also evident in the clinical validation studies from which different thresholds for CNB and RP had been established [5, 6]. These differences may be due to a number of factors including the sample collection methodology used, differences in the size of the sample tumour area, tumour heterogeneity and variability in Gleason scores. Those patient samples with assay scores falling at or within their respective reference intervals can be reliably reported within our CLIA laboratory.

**Table 2:** 95% reference intervals for the Metastatic Assay

	CNB			RP		
	Estimate	Lower 90% CI	Upper 90% CI	Estimate	Lower 90% CI	Upper 90% CI
Lower 95% Reference Interval	0.150	0.054	0.233	−0.214	− 0.311	− 0.127
Upper 95% Reference Interval	1.107	1.020	1.195	0.844	0.758	0.943

*Abbreviations: CI*= confidence interval, *CNB=* core needle biopsy, *RP=* radical prostatectomy

### Precision

**Fig. 4:**
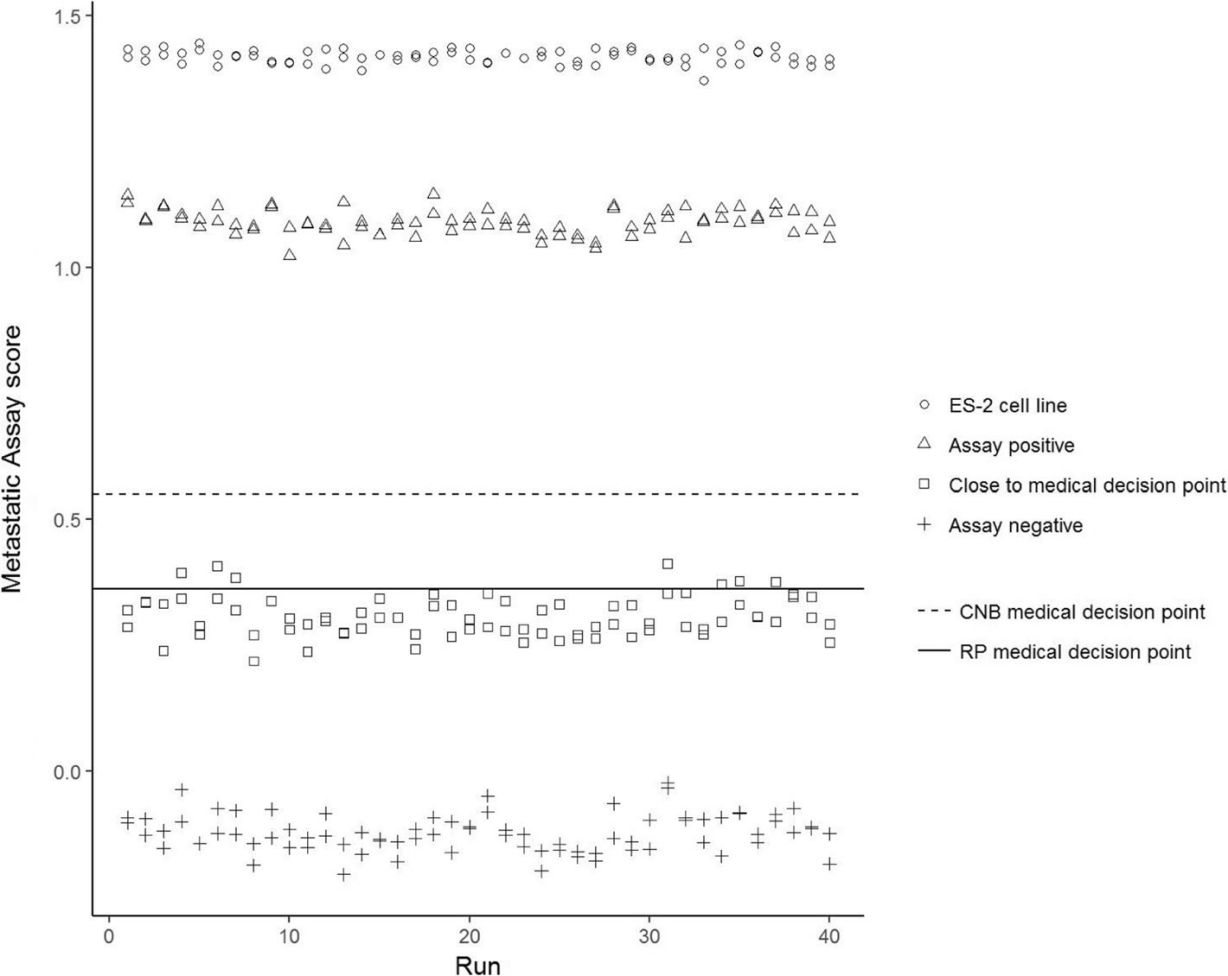
Results of duplicate runs (*n* = 40) of an ES-2 cell line sample, an Assay Positive (CNB) sample, an Assay Negative (RP) sample and a sample close to the medical decision point (RP) for precision evaluation

The repeatability and reproducibility of the assay was determined using one FFPE cell line (ES-2), representing high assay scores and three pooled clinical samples (assay positive (RP), assay negative (CNB) and one sample close to its sample specific medical decision point (RP)) (Fig. 4). All QC criteria were satisfied. Table 3 summarises the results obtained from the nested linear mixed model, displaying the model-based mean and the SD point estimates for all sources of variation, including the reproducibility of the assay (total SD), and the repeatability (within run SD). Following an assessment of misclassification the percentage of clinical samples estimated to change assay call due to assay imprecision was only 4.3, 95% CI (1.7, 7.5%). Conversely 95.7% of clinical sample scores were correctly classified.

**Table 3:** Analytical Precision

Sample	Model Mean	Day SD (95% CI)	Run SD (95% CI)	Equipment SD (95% CI)	Reagent Lot SD (95% CI)	Operator SD (95% CI)	Within Run SD (95% CI)	Total SD (95% CI)
ES-2 cell line	1.418	0 (0, 0.006)	0 (0, 0.002)	0 (0, 0.002)	0 (0, 0.002)	0 (0, 0.007)	0.014 (0.011, 0.015)	0.014 (0.012, 0.016)
Assay Negative	−0.123	0.010 (0, 0.024)	0.006 (0, 0.014)	0.014 (0, 0.022)	0.010 (0, 0.017)	0.016 (0, 0.021)	0.027 (0.022, 0.033)	0.038 (0.031, 0.048)
Medical Decision Point	0.307	0 (0, 0.022)	0.010 (0, 0.016)	0.010 (0, 0.016)	0.013 (0, 0.022)	0.015 (0, 0.020)	0.032 (0.026, 0.039)	0.040 (0.035, 0.053)
Assay Positive	1.090	0 (0, 0.014)	0.008 (0, 0.011)	0.008 (0, 0.012)	0.009 (0, 0.013)	0.007 (0, 0.009)	0.019 (0.015, 0.023)	0.025 (0.021, 0.031)

Model mean and standard deviation (SD) point estimates from nested linear mixed model. *CI* confidence interval

### Analytical sensitivity

In direct comparisons to the total RNA input of the Ovation FFPE WTA System amplification kit (50 ng), no statistically significant difference was found in the assay score at total RNA inputs of 25 ng and 100 ng. A significant difference in the assay score was observed for 12.5 ng (Table 4).

**Table 4:** Linear mixed effects model estimates comparing assay score at varying total RNA inputs

Comparison (ng)	Estimate	Standard Error	Lower 95% CI	Upper 95% CI	*p*-value
12.5 vs 50	0.0310	0.0150	0.0010	0.0600	0.0429
25 vs 50	0.0180	0.0150	−0.0120	0.0480	0.2311
100 vs 50	−0.0220	0.0150	−0.0520	0.0080	0.1403

*Abbreviations: CI* =confidence interval

Furthermore, a significant difference in the assay score was observed at a cDNA input of 0.88 μg in comparison to the nominal assay input (3.5 μg) while no significant difference was detected at cDNA inputs of 1.75 μg or 7 μg (Table 5).

**Table 5:** Linear mixed effects model estimates comparing assay score at varying total cDNA inputs

Comparison (μg)	Estimate	Standard Error	Lower 95% CI	Upper 95% CI	*p*-value
0.88 vs 3.50	0.0230	0.0070	0.0090	0.0370	0.0030
1.75 vs 3.50	0.0110	0.0070	−0.0040	0.0250	0.1370
7.00 vs 3.50	0.0002	0.0070	−0.0140	0.0140	0.9800

*Abbreviations: CI* =confidence interval

## Discussion

Prostate cancer is one of the most common malignancies in adult men in the developed world. Furthermore, patients often develop metastasis despite the initial success of primary treatment. An assay that stratifies patients based on metastatic risk would be a valuable tool for physicians to inform treatment decisions. The Metastatic Assay can quantify gene expression to identify a subgroup of primary prostate cancer patients that are at risk of metastatic disease recurrence. Incorporation of such a biomarker within clinical practice requires evidence of analytical validity to ensure decisions around patient management are based on precise and reliable data. The assay was therefore evaluated through a series of analytical studies and all specified acceptance criteria were met.

The use of CNB samples in the clinical diagnosis of prostate cancer is standard practice. Viable tumour material from CNB samples that can be used for molecular analysis is often limited and is further hindered by its fixation in formalin leading to a highly fragmented RNA template [23, 24]. The Metastatic Assay has been developed to utilise small amounts of tumour material from FFPE CNB and RP specimens. More specifically, the assay uses both 3′ end and random priming throughout the whole transcriptome making it suitable for amplification of degraded RNA. One of the challenges in analytically validating novel biomarkers is demonstrating the accuracy of an assay where no accepted standard exists. In this study accuracy was demonstrated by a 94.7% concordance between the development platform and the Xcel™ microarray, an FFPE optimized microarray containing probes targeting cancer specific content from multiple cancer types. Given the overlap of genes targeted by each of the two array types (*n* = 19,465), of which 70 are the Metastatic Assay genes, the high concordance is not surprising. Similarly there was a high level of agreement between the cDNA microarray and the Nanostring nCounter® platforms (94.2%) while a lower overall agreement with the RNA sequencing platform (87.9%) was noted. However, we believe that following optimization of assay parameters the Nanostring nCounter® and RNA sequencing platforms could be alternative platforms for delivery of the Metastatic Assay.

Assay precision and reproducibility was assessed over an extensive period of time (20 weeks) and estimated as a SD while taking into account all potential sources of variation (operator, day, reagent lot, equipment, run and within-run). The true interpretation of an assay’s performance is only meaningful when applied clinically. To test this we applied the quantified assay variation to clinical sample scores from the reportable range study where 95.7% of clinical samples were correctly classified after introducing the estimated level of assay imprecision into the assay scores. These results demonstrate a robust assay capable of reproducing results regardless of the influence of potential variability in the processing of clinical samples.

Use of a dilution series for determination of assay sensitivity of a microarray-based assay can be challenging due to the multiplex nature of the assay, the binary format of the microarray output and the potential influence of biochemical and instrumental noise on signal amplification. As such, assay LLOQ was determined by treating the total RNA as a single analyte and determining the influence of total RNA input on assay score. Despite yielding sufficient cDNA product (3.5 μg) to permit microarray analysis, an assay input of 12.5 ng of RNA (3.1 ng/μl) had a significant effect on assay score. Similarly, a significant difference in assay score was apparent when 0.88 μg of cDNA was applied to the microarray. With application of around one quarter of the manufacturer’s recommended total RNA and cDNA amounts this outcome is not surprising. Despite the multiple probe set design of the array aimed at improving sensitivity, this significant change in assay score is possibly due to a random distribution of the Metastatic Assay specific transcripts as a consequence of sample dilution [25, 26]. Nonetheless, the LLOQ of the assay input is at most 50 ng (12.5 ng/μl) and whilst the total RNA amount is appropriate it may be conservative. Significant consistency in assay score was demonstrated with 25 ng of total RNA. Therefore the Metastatic Assay may be suitable for use in a clinical setting where RNA yield from FFPE biopsies is limited. Furthermore, the microarray platform on which the assay has been developed has high gene content with probe sets targeting over 20,000 genes. This provides the opportunity to evaluate additional biomarkers in a research setting that may complement the prognostic utility of the assay, thereby maximising the diagnostic information from little sample material.

## Conclusion

The Metastatic Assay has been clinically validated to predict the risk of prostate cancer metastasis. The assay uses FFPE tissue from both radical prostatectomy and biopsies to generate an assay score and dichotomized call based on the presence or absence of metastatic like biology. This study provides evidence for the robustness and analytical reproducibility of the Metastatic Assay and further supports its potential use as a clinical tool for prostate cancer risk stratification.

## Additional files

Additional file 1: **Table S1.** *Metastatic Assay Gene List including Prostate Metastatic Assay gene weightings and bias.* **Table S2.** Summary of Clinical Characteristics of the patient samples used for analytical assessment. **Table S3.** A Metastatic Assay calls between the microarray and Nanostring nCounter® platforms. B Agreement in Metastatic Assay call between the microarray and Nanostring nCounter® platforms. **Table S4.** A Metastatic Assay calls between the microarray and RNA sequencing platforms. B Agreement in Metastatic Assay call between the microarray and RNA sequencing platforms. **Figure S1.** Scatter plot of Metastatic Assay scores between the microarray and Nanostring nCounter® platforms. Each data point represents the assay score of the same sample profiled on the microarray and Nanostring nCounter® platforms. **Figure S2.** Scatter plot of Metastatic Assay scores between the microarray and RNA sequencing platforms. Each data point represents the assay score of the same sample profiled on the microarray and RNA sequencing platforms. (DOCX 196 kb)

Additional file 2: Supplementary Methods. (DOCX 20 kb)
