# Supplementary material for: Analytical validation of a prognostic prostate cancer gene expression assay using formalin fixed paraffin embedded tissue
Source: BMC Med Genomics. 2018 Dec 27;11:125. doi: 10.1186/s12920-018-0442-y (PMC6307209; doi:10.1186/s12920-018-0442-y)
Supplement: Supplementary file 1 — Table S1. Metastatic Assay Gene List including Prostate Metastatic Assay gene weightings and bias. Table S2. Summary of Clinical Characteristics of the patient samples used for analytical assessment. Table S3. A Metastatic Assay calls between the microarray and Nanostring nCounter® platforms. B Agreement in Metastatic Assay call between the microarray and Nanostring nCounter® platforms. Table S4. A Metastatic Assay calls between the microarray and RNA sequencing platforms. B Agreement in Metastatic Assay call between the microarray and RNA sequencing platforms. Figure S1. Scatter plot of Metastatic Assay scores between the microarray and Nanostring nCounter® platforms. Each data point represents the assay score of the same sample profiled on the microarray and Nanostring nCounter® platforms. Figure S2. Scatter plot of Metastatic Assay scores between the microarray and RNA sequencing platforms. Each data point represents the assay score of the same sample profiled on the microarray and RNA sequencing platforms. (DOCX 196 kb) [file 12920_2018_442_MOESM1_ESM.docx]

**SUPPLEMENTARY TABLES AND FIGURES**

**Table S1** *Metastatic Assay Gene List* *including Prostate Metastatic Assay gene weightings and bias.*

| **Gene Name** | **Entrez Gene ID** | **Weight** | **Bias** |
| --- | --- | --- | --- |
| CAPN6 | 827 | -0.010899 | 4.440873 |
| THBS4 | 7060 | -0.009632 | 6.912586 |
| PLP1 | 5354 | -0.008886 | 4.383572 |
| MT1A | 4489 | -0.008681 | 6.747957 |
| MIR205HG | 406988 | -0.008279 | 7.215245 |
| SEMG1 | 6406 | -0.007935 | 4.230423 |
| RSPO3 | 84870 | -0.007296 | 4.293173 |
| ANO7 | 50636 | -0.007164 | 6.522548 |
| PCP4 | 5121 | -0.007139 | 7.621758 |
| ANKRD1 | 27063 | -0.006922 | 5.928315 |
| MYBPC1 | 4604 | -0.006845 | 4.574319 |
| MMP7 | 4316 | -0.006835 | 6.756722 |
| SERPINA3 | 12 | -0.006831 | 5.745462 |
| SELE | 6401 | -0.006810 | 5.977682 |
| KRT5 | 3852 | -0.006403 | 6.080494 |
| LTF | 4057 | -0.006400 | 6.497260 |
| KIAA1210 | 57481 | -0.006381 | 3.559966 |
| TMEM158 | 25907 | -0.006312 | 8.063421 |
| ZFP36 | 7538 | -0.006271 | 9.960827 |
| FOSB | 2354 | -0.006108 | 6.954936 |
| PCA3 | 50652 | -0.006102 | 5.262342 |
| TRPM8 | 79054 | -0.006060 | 4.865791 |
| PTTG1 | 9232 | 0.006017 | 4.712693 |
| LOC283194 | 283194 | -0.005950 | 4.980381 |
| PAGE4 | 9506 | -0.005837 | 7.073907 |
| STEAP4 | 79689 | -0.005685 | 8.105295 |
| TMEM178A | 130733 | -0.005647 | 7.594526 |
| CXCL2 | 2920 | -0.005598 | 8.928978 |
| HS3ST3A1 | 9955 | -0.005593 | 4.232782 |
| EYA1 | 2138 | -0.005581 | 5.504276 |
| RSPO2 | 340419 | -0.005563 | 3.922421 |
| PKP1 | 5317 | -0.005553 | 5.912186 |
| MUC6 | 4588 | -0.005522 | 6.640037 |
| PENK | 5179 | -0.005506 | 4.514855 |
| DEFB1 | 1672 | -0.005400 | 6.825491 |

**Table S1** **(cont’d)**

| **Gene Name** | **Entrez Gene ID** | **Weight** | **Bias** |
| --- | --- | --- | --- |
| SLC7A3 | 84889 | -0.005390 | 4.649004 |
| MIR578 | 693163 | -0.005355 | 5.087389 |
| PI15 | 51050 | -0.005264 | 4.858716 |
| UBXN10-AS1 | 101928017 | -0.005259 | 6.065878 |
| PDK4 | 5166 | -0.005249 | 4.174094 |
| PHGR1 | 644844 | -0.005208 | 5.183571 |
| SERPINE1 | 5054 | -0.005195 | 6.691866 |
| PDZRN4 | 29951 | -0.005147 | 4.752328 |
| ZNF185 | 7739 | -0.005105 | 6.900544 |
| ADRA2C | 152 | -0.005055 | 7.078377 |
| AZGP1 | 563 | -0.005018 | 8.191178 |
| TK1 | 7083 | 0.004966 | 5.581335 |
| POTEH | 23784 | -0.004961 | 4.824976 |
| KIF11 | 3832 | 0.004929 | 3.917669 |
| CLDN1 | 9076 | -0.004924 | 4.960283 |
| MIR4530 | 100616163 | -0.004908 | 10.536452 |
| MAFF | 23764 | -0.004901 | 8.497945 |
| ZNF765 | 91661 | -0.004862 | 3.976333 |
| CKS2 | 1164 | 0.004856 | 6.503981 |
| TCEAL7 | 56849 | -0.004856 | 4.819328 |
| PLIN1 | 5346 | 0.004831 | 4.629392 |
| SIGLEC1 | 6614 | 0.004773 | 5.503752 |
| FAM150B | 285016 | -0.004773 | 6.664595 |
| MFAP5 | 8076 | -0.004772 | 4.129177 |
| SFRP1 | 6422 | -0.004762 | 7.901262 |
| DUSP5 | 1847 | -0.004718 | 5.762678 |
| VARS2 | 57176 | 0.004675 | 5.223455 |
| ABCC4 | 10257 | -0.004664 | 5.230377 |
| SH3BP4 | 23677 | -0.004623 | 4.882708 |
| SORD | 6652 | -0.004573 | 8.958411 |
| MTERFD1 | 51001 | 0.004522 | 5.334199 |
| DPP4 | 1803 | -0.004506 | 4.659748 |
| AATBC | 284837 | 0.004502 | 4.905313 |
| FAM3B | 54097 | -0.004443 | 7.388071 |
| KLK3 | 354 | -0.004425 | 10.226441 |

**Table S2** Summary of Clinical Characteristics of the patient samples used for analytical assessment

| **Sample Type** |  | ***n*** | **%** |
| --- | --- | --- | --- |
|  | *CNB* | 60 | 50 |
|  | *RP* | 60 | 50 |
| **Clinical Site** |  | **n** | **%** |
|  | *IPCRC* | 24 | 20 |
|  | *Oslo* | 35 | 29 |
|  | *WCB* | 2 | 2 |
|  | *BCH (NI Biobank)* | 59 | 49 |
| **Patient Age (Years)** |  | **Median** | **Range** |
|  |  | 63 | 44 -74 |
| **Recurrence Event** |  | **n** | **%** |
|  | *Recurrence* | 45 | 38 |
|  | *Non-recurrence* | 66 | 55 |
|  | *Unknown* | 9 | 7 |
| **Time to Recurrence (Months)** |  | **Median** | **Range** |
|  |  | 34 | 2 - 121 |
| **PSA (ng/ml)** |  | **Median** | **Range** |
|  |  | 14 | 4.1 - 438 |
| **Gleason** |  | ***n*** | **%** |
|  | *6* | 7 | 6 |
|  | *7* | 38 | 32 |
|  | *8-10* | 64 | 53 |
|  | *Unknown* | 11 | 9 |
| **T-stage** |  | ***n*** | **%** |
|  | *2* | 26 | 22 |
|  | *3* | 62 | 52 |
|  | *4* | 8 | 7 |
|  | *Unknown* | 24 | 19 |

*PSA* prostate specific antigen, *CNB* core needle biopsy, *RP* radical prostatectomy, *IPCRC* Irish Prostate Cancer Research Consortium Biobank, *WCB* Wales Caner Bank, *BCH* Belfast City Hospital

**Table S3A** Metastatic Assay calls between the microarray and Nanostring nCounter^®^ platforms.

|  | | **Nanostring nCounter**^®^ **Platform** | | |
| --- | --- | --- | --- | --- |
|  |  | **Assay Positive** | **Assay Negative** | **Total** |
| **Microarray Platform** | **Assay Positive** | 26 | 3 | 29 |
|  | **Assay Negative** | 1 | 39 | 40 |
|  | **Total** | 27 | 42 | 69 |

**Table S3B** Agreement in Metastatic Assay call between the microarray and Nanostring nCounter^®^ platforms

| **Percent Agreement** | **Estimate (%)** | **95% confidence interval (%)** |
| --- | --- | --- |
| **Overall** | 94.2 | 85.8 – 98.4 |
| **Positive** | 89.7 | 72.6 – 97.8 |
| **Negative** | 97.5 | 86.8 – 99.9 |

**Table S4A** Metastatic Assay calls between the microarray and RNA sequencing platforms.

|  | | **RNA Sequencing Platform** | | |
| --- | --- | --- | --- | --- |
|  |  | **Assay Positive** | **Assay Negative** | **Total** |
| **Microarray Platform** | **Assay Positive** | 22 | 5 | 27 |
|  | **Assay Negative** | 3 | 36 | 39 |
|  | **Total** | 25 | 41 | 66 |

**Table S4B** Agreement in Metastatic Assay call between the microarray and RNA sequencing platforms

| **Percent Agreement** | **Estimate (%)** | **95% confidence interval (%)** |
| --- | --- | --- |
| **Overall** | 87.9 | 77.5 – 94.6 |
| **Positive** | 81.5 | 61.9 – 93.7 |
| **Negative** | 92.3 | 79.1 – 98.4 |

**
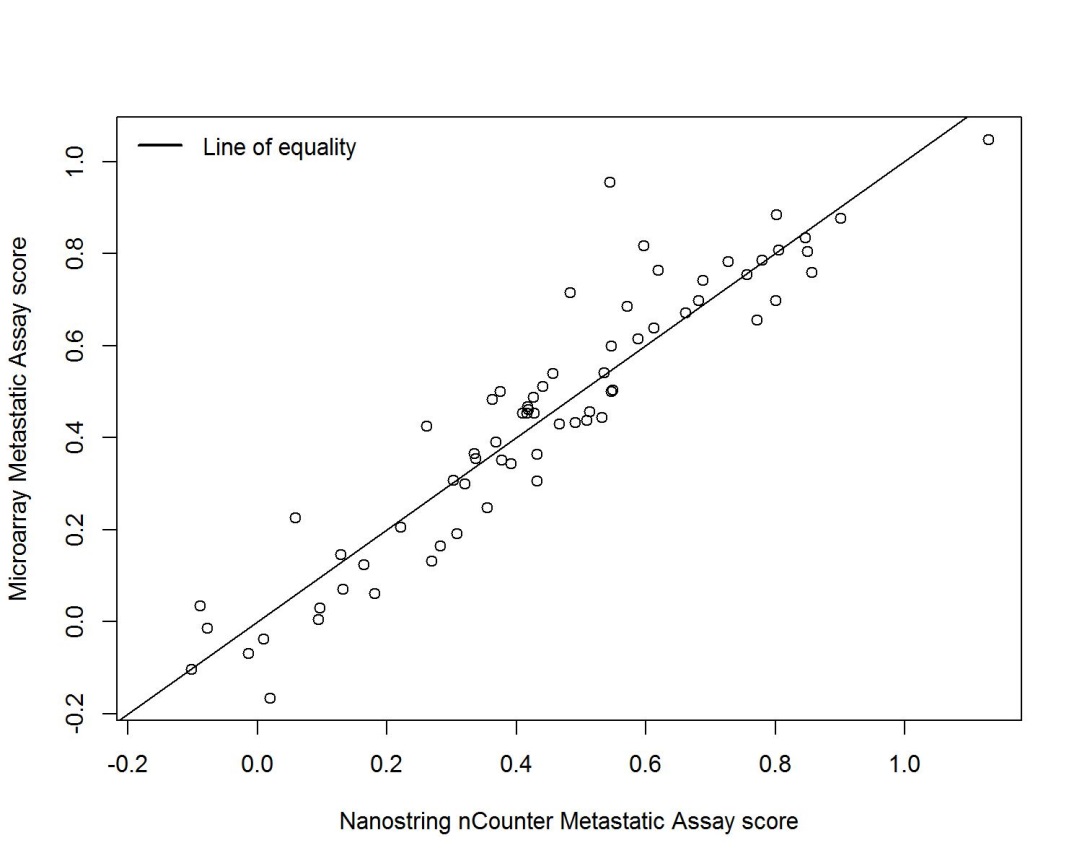
**

**Figure S1** Scatter plot of Metastatic Assay scores between the microarray and Nanostring nCounter^®^ platforms. Each data point represents the assay score of the same sample profiled on the microarray and Nanostring nCounter^®^ platforms

*
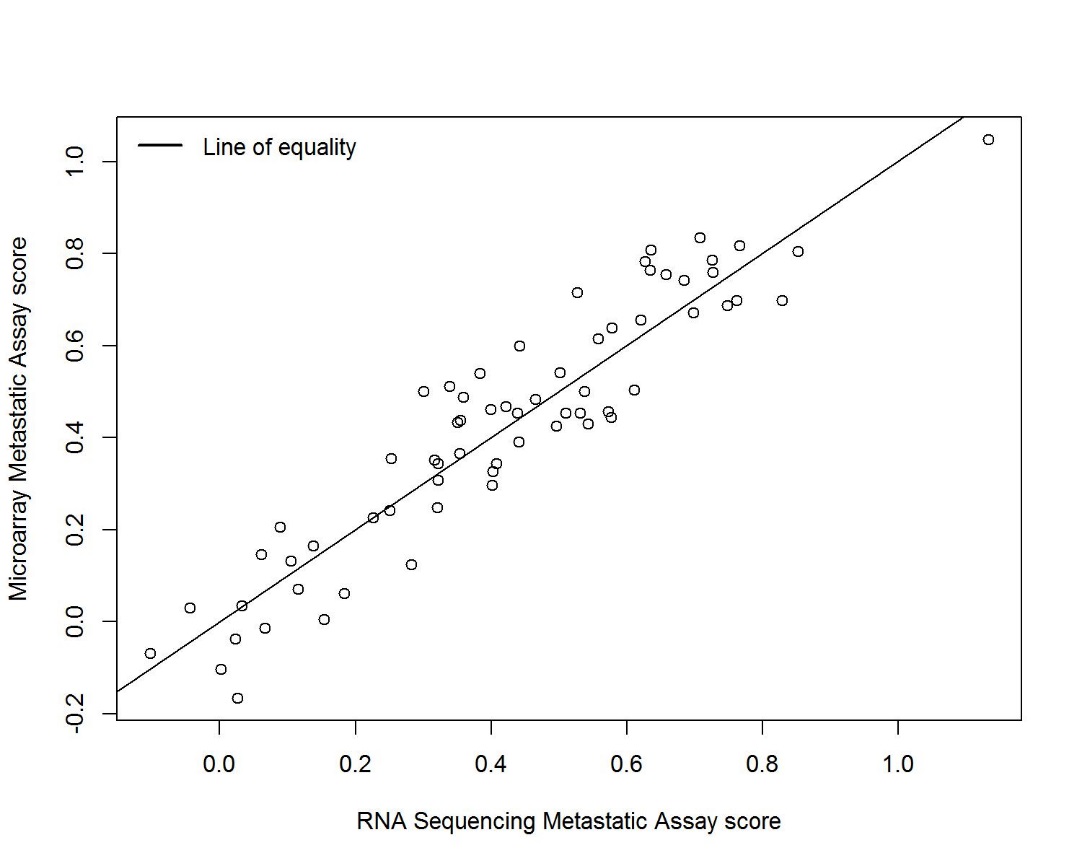
*

**Figure S2** Scatter plot of Metastatic Assay scores between the microarray and RNA sequencing platforms. Each data point represents the assay score of the same sample profiled on the microarray and RNA sequencing platforms
