# Supplementary material for: Analytical validation of a prognostic prostate cancer gene expression assay using formalin fixed paraffin embedded tissue
Source: BMC Med Genomics. 2018 Dec 27;11:125. doi: 10.1186/s12920-018-0442-y (PMC6307209; doi:10.1186/s12920-018-0442-y)
Supplement: Supplementary file 2 — Supplementary Methods. (DOCX 20 kb) [file 12920_2018_442_MOESM2_ESM.docx]

**SUPPLEMENTARY METHODS**

***Sample Information***

Samples were selected for analytical assessment with an equal split between CNB (*n*=60) and RP (*n*=60), totalling 120 unique patients. Samples ranged across four clinical sites with the majority of samples from the Belfast Health and Social Care Trust (BCH/NI Biobank, followed by 29% from Oslo University Hospital, 20% the Irish Prostate Cancer Research Consortium (IPCRC) and 2% from Wales Cancer Bank (WCB). Overall, 38% of patients experienced a known recurrence wither biochemical or metastatic, 55% had no recurrence and 7% were unknown. Median time to recurrence event was 34 months. In total 6% of patients had a high Gleason score <7, with 32% having a Gleason 7, 53% patients >7 and the remaining patients unknown. The majority of patients (52%) had a T-stage of T3, with 22% T2, 7% T4 and 19% unknown. Median PSA levels were 14 ng/ml and median age of 63 years (See Supplementary Table 1, Additional File 1).

***RNA Extraction of Prostate Cancer samples***

Total RNA was extracted from 70 FFPE samples using the Roche High Pure RNA Paraffin kit (Roche, Basel, Switzerland) as outlined in the main text Materials and Methods. Total RNA were assessed for concentration (ng/µl) using the Nanodrop spectrophotometer (ThermoFisher, Santa Clara, CA) and quality (DV_200_) using the Agilent 2100 Bioanalyzer (Agilent Technologies, Santa Clara, CA, USA).

***Nanostring Profiling of Prostate Cancer samples***

50 ng of total RNA from 70 samples (CNB=45; RP=25) was combined with reporter and custom codesets designed to target to target each of the 70 Metastatic Assay transcripts. The nCounter probesets were designed and synthesized by NanoString Technologies. Each probe pair was made complementary to a 100-base region of the target RNA sequence and designed to maximize coverage of all possible gene isoforms. . All samples were hybridised overnight for 18 hours at 65^o^C. Using the Nanostring PrepStation, all samples were added to the nCounter^®^ cartridge followed by image capture and data acquisition on the nCounter^®^ digital analyser. Measurements were taken at high resolution with 280 fields of view (FOV) per flow cell.

***Sequence Profiling of Prostate Cancer samples***

100 ng of total RNA from 66 samples (CNB=40; RP=26), with DV_200_ scores greater than 30%, was used to generate transcriptome capture based libraries with the Roche NimbleGen SeqCap RNA Targeted Enrichment kit ( Roche, Basel, Switzerland) in conjunction with a rapid capture custom probe set designed to capture the Metastatic Assay targets. All assay probes were designed and manufactured by Roche Life Sciences. Each probe was designed against the UCSC Human Genome 19 reference to target the whole gene sequence. Library quality and yield was assessed using an Agilent 2100 Bioanalyzer (Agilent Technologies, Santa Clara, CA, USA). Sequencing was performed on a MiSeq sequencing instrument (Illumina Inc.) with 2 × 76 cycles, using the Illumina MiSeq kit V3 sequencing chemistry according to the manufacturer’s instructions. Images from the instrument were processed using the manufacturer’s software to generate FASTQ sequence files.

***Data preparation & Quality Control (QC)***

Microarray Data

Samples were pre-processed using the Robust Multi-Array (RMA) average methodology. The QC assessment comprised a combination of the following quality metrics including array image analysis, GeneChip QC, principal components analysis (PCA) and intensity distribution analysis. Array data was examined to identify any image artefacts. As part of the GeneChip QC, percent present (%P), average signal absent, scale factor, average background and raw Q were all assessed. Samples with a %P<15% were deemed a QC fail. Hotelling T^2^ and residual Q method was used to identify sample outliers at the expression level within the PCA analysis. Finally, Kolmogorov-Smirnov statistic was used to examine the intensity distribution of the samples and identify outliers.

Nanostring Data

Nanostring nSolver analysis software (v2.6) was used to assess data quality. The following Nanostring default metrics were assessed: Image quality, Binding density, Positive control linearity and Positive control limit of detection. A single sample was classified as QC fail due to QC flags on binding density, positive control linearity and positive control limit of detection. Nanostring count data was used for the Metastatic Assay scoring.

Sequencing Data

FASTQ files were assessed using FASTQC app (v1.0.0) and read and sample quality metrics were assessed. Quality was verified using the following parameters: average Phred quality score, average GC content and total number of paired reads. Reads were mapped to the human genome hg19 using TopHat 2 aligner and gene expression estimated using Cufflinks 2. The resulting FPKMs (Fragments Per Kilobase of transcript per Million mapped reads) were used to generate Metastatic Assay scores.

***Metastatic Assay Score generation***

Nanostring and Sequencing Data

Initially a transformation was applied to Nanostring nSolver data (Log_2_(Counts+m)) and FPKM sequencing data (Log_2_(FPKM+n)), where m (=1) and n (=1.14) are constants applied to scale data > 0 prior to Log_2_ transformation. The transformed data used to generate Metastatic Assay scores using Partial Least Squares^1^ (PLS) regression analysis, where refined platform specific parameters were defined. Pearson correlation coefficient was used to determine association of Metastatic Assay scores between development and Nanostring or sequencing platforms.

***Assessment of Analytical Accuracy using Linear Bias Correction***

A Bland-Altman plot (Figure 2A) and scatter plot (Figure 2B) were used to visualise the bias between the assay scores on the Prostate DSA and Xcel^TM^ array platforms. The assumption of a constant bias in Metastatic Assay score between the platforms was violated (*p* < 0.0001) (Figure 2A). To account for the non-constant bias between the platforms the method of alternating regressions was implemented using the MethComp R package to obtain a linear relationship between the platforms whilst accounting for the non-constant bias between the platforms and modelling replicate measurements per sample explicitly [27]. The linear relationship between the platforms can be described by a regression line with intercept equal to -0.2617 and slope equal to 1.2638. After applying the linear bias correction the estimated bias in Metastatic Assay score between the platforms was reduced to approximately 0, with 95% of differences in the range of -0.091 to 0.090 (Figure 2C). Estimates of the variance components and bias underlying the comparison of the two platforms were also derived using the MethComp R package [27]. The Coefficient of Individual Agreement (CIA) was derived using the appropriate variance components from the regression model resulting in a CIA value of δ = 0.78 [28] satisfying the predefined criteria for agreement (δ = 0.78 < 1.24).

**References**

[27] Carstensen B, Gurrin L, Ekstrom C, Figurski M: MethComp: Functions for Analysis of Agreement in Method Comparison Studies 2015, R package version 1.22.2. <https://CRAN.R-project.org/package=MethComp>

[28] Barnhart HX, Andrzej SK, Haber MJ: Assessing individual agreement. J Biopharm Stat 2007, 17(4): 697-719.
